# Renal Trauma: A 4-Year Retrospective Review of Injury Severity, Treatment Approaches, and Outcomes from a Polish Trauma Center

**DOI:** 10.3390/clinpract15040067

**Published:** 2025-03-21

**Authors:** Michał Kasperczak, Anita Zaręba, Karolina Pawłowska-Kasperczak, Filip Kasperczak, Monika Zaręba, Andrzej Antczak

**Affiliations:** 1 Department of Urology, J. Struś Hospital in Poznań, Szwajcarska 3, 61-285 Poznan, Poland; kara040693@icloud.com (K.P.-K.);; 2 Department of Urology, Holycross Cancer Centre in Kielce, 25-734 Kielce, Poland; anitazareba7@o2.pl; 3 Collegium Medicum, Jan Kochanowski University, 25-369 Kielce, Poland

**Keywords:** renal injury, blunt trauma, penetrating trauma, management of injury

## Abstract

**Background:** The management of renal injuries in hemodynamically stable adult patients is moving toward more conservative methods, even in cases of severe grade and/or penetrating trauma. The objective of this study was to analyze the patterns of injury, management, and complications in renal trauma patients at a Polish trauma center. **Methods:** Patients diagnosed with renal trauma at the trauma center between January 2019 and December 2023 were identified based on the ICD-10 codes. The information was gathered from digitalized medical records, while imaging data were classified by Radiologists. **Results:** During a period of 4 years, a total of 81 patients with renal trauma were admitted to the trauma center. 76% of these patients were males, with a mean age of 44.61 ± 16.8 years. The most common concomitant conditions, both among men and women, included retroperitoneal hematoma, rib fractures, as well as chest and lung injuries. Surgical intervention within 8 h of admission was mainly performed on patients with grade IV and V kidney damage, which included a total of 22 people. In deferred treatment, 31 patients underwent surgical intervention. **Conclusions:** Hemodynamically stable patients, even with penetrating and/or high-grade blunt trauma, were mostly managed non-operatively, with a low rate of complications.

## 1. Introduction

Injuries are a common cause of disability and death worldwide, with trauma defined as a physical injury or a wound to living tissue caused by an extrinsic agent [1]. The number of multi-organ injuries increases every year, mostly due to widespread motorization. It is the third most common cause of death in the whole human population, and the second among people under 40 years of age [2]. Such injuries are most often the result of traffic accidents and falls from heights, which account for over 80% of such injuries [3,4,5].

Among multi-organ trauma cases, kidney injuries account for 1–5% [6,7,8]. In the United States, kidney injuries occur at a rate of approximately 4.9 cases per 100,000 people per year. Death due to multiple-organ injury occurs twice as often in men as women [1,9]. The morbidity and mortality rates vary with the severity, associated injuries, and the treatment of choice [10]. In European Union countries, injuries remain a major medical challenge. According to EUROSAT data from 2016, trauma was the fifth most common cause of death in the European population. In this report, injuries to the urogenital system constituted 10% of trauma cases, of which 29.66% were attributed to kidney injuries [1].

Kidney injuries are categorized as blunt and penetrating [11,12]. Blunt trauma can be a result of motor vehicle accidents (MVA), falls, sports injuries, assaults, and pedestrian accidents [9]. It most often results in the direct crushing of the kidney or hilum, and less frequently, in the detachment of the vascular structures of the hilum or the ureteropelvic junction (UPJ) [1]. Blunt trauma accounts for up to 95% of renal injuries [13]. In turn, penetrating injuries can be caused by stabbing or gunshot wounds, and may result in the disruption of parenchyma, blood vessels, or the calyceal system [14,15].

Current treatment standards for kidney trauma are strictly defined by the EAU and AAST guidelines. Diagnosis is performed using computed tomography (CT), and minimally invasive techniques such as angiography and endovascular interventions, which are used to determine the degree of injury according to the classification proposed by the American Association for the Surgery of Trauma (AAST) [9,16]. The AAST renal injury scale has been validated, accurately predicting the risk of and the need for procedural intervention [9,17,18]. Most grade I–IV injuries are currently treated conservatively [19,20], with the treatment of high-grade injuries depending on the type of trauma (blunt, penetrating), active bleeding, and follow-up imaging tests [1]. Nonoperative management was reported to fail only in 6.5% of patients suffering from renal injury [21].

Hence, the study aimed to assess the course and treatment of kidney injury in men and women, taking into account the description of renal trauma characteristics and treatment modalities treated at the Department of Urology in Poznan.

## 2. Materials and Methods

This retrospective study was carried out based on the data obtained from the Department of Urology in Poznan from 2019 to 2023. It included all adult patients with renal injuries, as diagnosed and graded using a CAT scan. The data, including patient age, gender, mechanism of injury, site of injury, grade of injury, vital signs, laboratory and imaging data, complications, treatment plan, and the length of hospital stay, were collected from a chart review. Renal injury was diagnosed based on medical history and symptoms, as well as physical and radiological examinations. The degree of kidney injury was qualified according to the AAST guidelines. The AAST renal injury scale was updated in 2018 [22]. However, all references included in this text are based on the AAST 1989 renal injury scale, as the 2018 injury scale does not outperform the previous grading system in predicting bleeding and the need for treatment intervention. It does not impact the validity of the current recommendations [23].

Differences in frequency distributions in categories of particular features were compared between the sexes using Pearson’s chi-squared test. Differences in means between two independent groups were assessed using the *t*-test for independent groups. Differences between more than two groups were assessed based on the one-way analysis of variance. The level of significance was assumed at a *p*-value below 0.05 (*p* < 0.05).

Furthermore, to control the initial results for associated injuries and co-morbidities, a comprehensive analysis was performed to determine the isolated effects of particular factors. A logistic regression model enhanced by LASSO (Least Absolute Shrinkage and Selection Operator) was employed. The initial LASSO logistic regression, which included a wide array of categorical variables—ranging from demographic factors like age and gender to specific injury types and comorbidities—served to pinpoint the most predictive variables by penalizing the less significant ones towards zero, thus not only refining the model for enhanced interpretability but also addressing potential overfitting issues. Subsequently, a simplified logistic regression model was developed, focusing on the predictors identified by LASSO as having substantial effects.

## 3. Results

The study comprised a total of 81 patients (62 male and 19 female). In the first stage of the study, a descriptive analysis of the study group was compiled, with simple comparisons between the frequencies of particular factors and conditions between the sexes and renal grade injuries. The average age of male and female patients was similar, at 44.61 years and 45.63 years, respectively. Among patients with kidney trauma, the majority were men (76.5%), who suffered severe kidney injuries significantly more often. No statistically significant differences were observed in the degree of severity of kidney injury in women. Blunt kidney trauma was the most common mechanism of injury in both men and women. Blunt injuries were characterized by varying degrees of kidney damage, and the differences turned out to be statistically significant. Both among men and women, the most common associated injuries included: retroperitoneal hematoma, rib fractures, and chest and lung injuries. In turn, patients were least commonly affected by urinary bladder, thoracic aorta, and stomach injuries. There was no statistical difference in the incidence of associated injuries between groups with different degrees of kidney damage (Table 1).

During the initial laboratory assessment, it was observed that the highest percentage of patients had leukocytosis, as 66 people (81.4%), including 51 men and 15 women, had WBC >11.0 10^9^/L. A small percentage of patients (4.9%) showed general symptoms of shock, assessed according to three criteria: tachycardia, oliguria, and systolic blood pressure below 90 mmHg. In turn, 28% of patients were given a GCS score of ≤8. Differences in the initial laboratory results between patients with various degrees of kidney damage turned out to be statistically insignificant. Due to the relatively young patient age, a small percentage of patients had any relevant co-morbidities (20.99% HT, 17.28% DM, 12, 35% CKD) (Table 2).

When assessing the type of medical treatment based on gender and the severity of trauma, it was found that the most common choice in both sexes was conservative treatment within the first 8 h of admission (72.84%) and in the final treatment (61.78%). Statistically significant differences were found between the severity of the injury and medical treatment, i.e., surgical or conservative treatment. Surgical treatment was more often undertaken in severe trauma and conservative treatment in minor and moderate injuries. The most frequently observed complications during hospitalization in both sexes were acute respiratory distress syndrome (39%), pneumonia (21%), and sepsis (16%). There were no statistically significant differences between the frequency of complications during hospitalization and gender and severity of injury (Table 3).

It is worth noting that, in all grades of kidney damage, patient survival was higher than mortality. In all distinct/separated groups, patient survival was over 80%. Nonetheless, the survival rates are not directly comparable between grades, due to the potential of being influenced by a range of associated factors.

To account for the combined influence of all of the concomitant injuries and co-morbidities, as well as to indicate the individual factors with the biggest effects on the analyzed parameters, a logistic regression analysis was conducted. The statistically significant results of the analysis, conducted separately for laboratory results and complications, were presented in Table 4 and Table 5. Due to a large number of factors and conditions, all non-significant results were omitted from the tables for clarity and readability.

The severity of the renal injury, particularly being categorized as “major”, was found to negatively impact the likelihood of maintaining hemoglobin levels above 10 g/dL. Male gender significantly increased the probability of higher hemoglobin levels post-injury. Age also negatively influenced hemoglobin levels, indicating a higher chance of lower hemoglobin levels in older patients. Furthermore, certain injury types, such as spleen injuries and injuries of the pancreas, were associated with lower probabilities of maintaining higher hemoglobin levels, possibly due to the nature of these injuries or their complications.

When it comes to creatinine levels over 110, a higher risk of their occurrence was attributed to patients with associated injuries of the pelvis and spleen, as well as prior presence of chronic kidney disease (CKD). CKD was also associated with an increased risk of glomerular filtration rate (GFR) <60, a parameter that also occurred more frequently in patients with associated injuries of the ileum. Moreover, the risk of systolic blood pressure (SBP) <90 increased in patients with associated injuries to the thoracic aorta.

When it comes to patient outcome complications, the presence of a lower extremity injury significantly impacted the risk of pneumonia development, while pancreatic injuries were significantly more present in patients that did not survive treatment. Finally, the prior presence of CKD significantly increased the risk of urinary tract infection and sepsis.

## 4. Discussion

Blunt trauma constitutes the predominant form of injury in countries with well-developed road transport systems and restricted access to firearms [13]. Similarly, in the group of patients in this study, as many as 86.42% of people had blunt injuries, and 13.58% had iatrogenic, stab, and gunshot injuries. Blunt trauma results from road traffic accidents/trauma, falls, sports injuries, and assaults [9]. In contrast, penetrating injuries, typically caused by stab and gunshot wounds, tend to be more severe and less predictable compared to blunt traumas [24]. The abdomen is the third most injured body region, and urogenital trauma comprises approximately 10% of all abdominal injuries [25].

The AAST classification is the most used assessment for the severity of kidney damage [26]. The injury level indicates the condition’s seriousness, directs treatment approaches, and assists in predicting outcomes. The scale is updated on an ongoing basis. In 2006, Buckley and McAninch proposed a revised grade IV, including the collecting duct system and segmental arterial and venous injuries, with grade V, including only hilum injuries [27]. In 2015, Chiron et al. also suggested updating the grade IV injury (perirenal hematoma >3.5 cm, contrast media extravasation) [28]. The last update from 2018 is presented in Table 1 in the methodology chapter. It does not differ from previous grading systems in predicting bleeding and the need for therapeutic intervention, and it does not affect the validity of current recommendations [23].

Hemoperitoneum, bleeding within the peritoneal cavity, is determined by FAST (Focused Assessment Sonography in Trauma) during the initial evaluation of a critically injured patient. Nevertheless, its limited sensitivity, reliance on operator skill, vague delineation of injuries, and inferiority compared to computerized tomography scans suggest it could serve as a secondary diagnostic tool [29,30,31].

In recent years, Computerized Tomography (CT) technology has undergone exponential advancements, enabling the detailed identification of vascular and parenchymal injuries, and collecting systems with unprecedented precision [32]. CT has established itself as a gold standard for the diagnosis of renal injuries, and its use is the recommended diagnostic method if the patient is hemodynamically stable [33,34]. On the other hand, CAT sensitivity is around 93%, with a specificity of 100%. In determining the non-operative management of renal trauma, it is not necessary to routinely perform serial CAT scans. Instead, the decision regarding repeat CAT scans should be personalized based on the injury severity, the clinical evolution of the patient, and the presence of associated abdominal injuries [35]. CAT scans must be performed on patients with a fever, unexplained decreased hematocrit, or significant flank pain. Repeat imaging is recommended in high-grade injury and in penetrating trauma two to four days after trauma to minimize the risk of missed complications [1]. Regarding renal trauma, MRI and CT have comparable diagnostic accuracy [36,37]. However, in cases of acute trauma, MRI is not a feasible technique due to its logistical limitations.

Historically, the most frequent injuries have been grade I (28%), followed by grade II (26%), grade III (19%), grade IV (18%), and grade V (9%). In recent years, there has been a noticeable shift in the clinical management of renal injury cases towards conservative, non-surgical approaches, especially among hemodynamically stable patients. Our findings corroborate these results [38]. Most injuries can be treated non-operatively with effective organ preservation, especially grade I–III [19,39]. Although the treatment of grade IV and V injuries remains a subject of debate, a review by Sujenthiran et al. concluded that conservative management is the best first-line option in stable patients for treating high-grade renal injuries in terms of safety and effectiveness. This approach is associated with a higher percentage of kidney preservation, a shorter hospitalization period, and a lower incidence of complications compared to open surgical exploration [19,40]. McGuire et al. showed that a grade V injury and the need for a platelet transfusion necessitate immediate surgical intervention [41].

Similarly, Yang et al. reported that the severity of injury and the degree of renal damage are the main predictors of surgical intervention [42]. Nephrorrhaphy, the most utilized technique, enables renal reconstruction in many cases. Partial nephrectomy may be required when the tissue viability is compromised, while total nephrectomy becomes necessary if repair is unattainable [43]. Exploratory and therapeutic laparoscopy can be employed for lesion staging and, if needed, for repair or nephrectomy. However, this approach is not recommended in hemodynamically unstable patients [43]. Our research aligns with these reports - surgical intervention was performed mainly on patients with grade V kidney damage (21 people) and grade IV (1 person) within 8 h of admission. In the deferred treatment group, thirty-one patients underwent surgical intervention, including eight people with grade V kidney damage and one with grade IV kidney damage. Both in surgical intervention within 8 h of admission and deferred treatment, nephrectomy prevailed, i.e., 63.64% and 66.67%, respectively.

The widespread adoption of angioembolization for addressing active bleeding complications has introduced an intermediate approach, bridging traditional surgery and non-operative management [44]. Angioembolization demonstrates efficacy and safety in managing stable grade III–IV blunt renal trauma. While grade I–II blunt renal injuries rarely necessitate renal angioembolization, its potential benefits in a subset of hemodynamically unstable or grade V patients are not firmly established. However, outcomes remain favorable for stable patients with moderate to high-grade renal trauma [45]. In the current study, embolization was performed in 27.27% of cases within 8 h of admission and in 11.11% undergoing deferred treatment. However, renography was performed in 9.09% of immediate surgery cases and in 11.11% of patients subjected to delayed treatment. Additionally, areas of bleeding were sutured in one patient undergoing deferred treatment. The conservative treatment of patients with severe kidney damage was provided to 19.75% of patients within the first hours of admission and 9.88% of patients in deferred treatment.

Another notable factor predicting the necessity of surgical intervention is the presence of retroperitoneal hematoma, which, in our study, occurred in all patients who underwent surgery within 8 h of admission and in delayed treatment. Additionally, the frequency of other injuries, particularly chest injuries and rib fractures, plays a significant role. Hardee et al. found that intravascular extravasation and a perinephric hematoma ≥3.5 cm were associated with a need for intervention; however, medial laceration was not [46]. Generally, the choice of treatment approach for blunt injuries depends on factors such as the anatomical location of the hematoma, the presence of visceral injury, and the hemodynamic stability of the patients [47].

The percentage of deaths among patients treated surgically turned out to be approximately two times lower than that in patients treated conservatively, i.e., 10.34% and 20.98%, respectively. It should be emphasized that 9 out of 13 deceased patients treated conservatively had a high- or medium-grade kidney injury.

The number of complications during hospitalization among patients treated surgically and conservatively was similar and included, respectively, dialysis (9.68%; 14%), acute kidney injury (9.68%; 14%), and urinary tract infection (10%; 6.45%), pressure ulcers (9.68%; 14%). Among patients treated surgically, sepsis (16.13%; 2.00%) and nosocomial pneumonia (16.13%; 6%) occurred more often than among those treated conservatively. In turn, acute respiratory distress syndrome was observed much more frequently in patients treated conservatively (62%) than in the second group (25.81%), and the same applied to deep vein thrombosis (18% and 9.78%, respectively). Therefore, this study did not confirm that surgical intervention in the case of kidney damage is associated with a reduction in the number of complications. Kuan et al. observed a dialysis requirement risk of 0.46% following renal damage, with ages over 40 and increasing AAST grade correlating with dialysis necessity [48]. Bozeman et al., in a series involving 26 patients with severe grade IV–V renal damage, found no statistically significant differences in morbidity between operative and nonoperative groups [38]. Conservatively handled patients had lower morbidity according to Altman et al., who reported lower morbidity in conservatively managed patients [49]. Chow et al. noted a 94.5% success rate in the conservative treatment of high-grade renal injuries (IV–V), surpassing outcomes associated with surgical intervention [50].

The length of hospitalization was slightly longer among patients treated conservatively, and amounted to 19.3 days, while in patients undergoing surgical intervention, it was 15 days. There was no notable disparity in hospital length of stay between conservative and surgical approaches for managing high-grade renal trauma [51].

The most frequent early complications occurring within one month, include urinary fistula, hypertension, perinephric abscess, infection, hemorrhage, and urinoma. Delayed complications include bleeding, calculus development, persistent pyelonephritis, hypertension, AVF, hydronephrosis, and pseudo-aneurysms. In cases where bleeding poses a risk to life, the recommended course of treatment is elective angiographic embolization. Percutaneous drainage is the first line of treatment for forming perinephric abscesses [1].

There is currently no conclusive evidence regarding the optimal timing for resuming regular activity after renal trauma. However, as a general guideline, it is recommended to observe bed rest or limit activity until gross hematuria has resolved [52,53]. Returning to practice sports activities after a minor or moderate renal injury may happen within 2 to 6 weeks from the injury, while severe injuries may require longer periods (6 to 12 months) [54,55]. Generally, sports activities should be avoided until microscopic hematuria is resolved [54,55]. As part of outpatient care, renal scintigraphy is typically scheduled 2–3 months after a renal injury. It is important to note that the risk of diminished kidney function tends to rise with the severity of the injury grade [56].

The limitations of this study are its retrospective nature and the lack of long-term follow-up of renal function in patients treated conservatively. Furthermore, the study was based on a relatively small sample size regarding the number of individual injury-associated factors, leading to limited statistical power when analyzing subgroups, and often effectively preventing more detailed multivariate analysis. Additionally, we acknowledge that our center’s decision-making process for managing patients with renal trauma is complex and involves multiple healthcare providers. This could introduce bias into our study due to variations in individual practice patterns and preferences, which should be considered when interpreting the results. Furthermore, our study is conducted at a single center, which may limit the generalizability of our findings to other settings. It is important to note that the most frequent causes of renal trauma in our area may not be representative of different regions, potentially introducing bias into our study findings. We recognize the need for a better description of the predominant causes of renal trauma in our local area to address this limitation. Nonetheless, our study still holds significance as one of the pioneering studies or possibly the sole study of this type based on the Polish population, contributing to its uniqueness. This highlights the importance of our research in providing insights specific to our region.

## 5. Conclusions

This study reveals the rarity of penetrating trauma cases at our hospital, with most blunt traumas attributed to falls, MVAs, and cycling incidents. Our findings support the notion that even severe blunt injuries and stable penetrating injuries present favorable outcomes after non-operative management. The goal of kidney trauma treatment is to determine the severity of kidney damage and minimize complications accurately. In the case of moderate- and high-grade injuries in stable patients, diagnostics and follow-up should be extended to select the most optimal treatment method.

## Figures and Tables

**Table 1:** Demographic, mechanism of injury, associated injuries of patients who have sustained blunt renal trauma according to AAST injury grades (three categories), and sex. ^1^—*t*-Student test for independent sample; ^2^—one-way analysis of variance; ^3^—Pearson’s chi-square test.

		Sex			AAST Injury Grades			
	Overall	Male	Female	P Level	Minor N = 23	Moderate N = 21	Severe N = 37	P Level
**Age; mean (SD)**	44.9 (18.2)	44.61 (16.8)	45.63 (22.9)	n.s.	44.91 (15.1)	41.33 (20.0)	46.81 (19.1)	n.s.
**Sex; n (%)**		62 (76.5)	19 (23.5)					
Male					18 (29.0)	14 (22.6)	30 (48.4)	0.4486 ^1^
Female					5 (26.4)	7 (36.8)	7 (36.8)	n.s.
**Mechanism of injury** **; n (%)**								
Blunt wound	70 (86.42)	54 (77.1)	16 (22.9)					0.0230 ^2^
Bullet wound	4 (5.00)	4 (100)	0 (0)	n.s.	23 (32.9)	19 (27.1)	28 (40.0)	n.s.
Iatrogenic would	1 (1.00)	1 (100)	0 (0)					n.s.
**Associated injuries; n (%)**								
Chest	52 (64.20)	40 (76.92)	12 (23.08)	n.s.	18 (34.62)	13 (25.00)	21 (40.38)	n.s.
Rib fracture	52 (64.20)	40 (76.92)	12 (23.08)	n.s.	18 (34.62)	13 (25.00)	21 (40.38)	n.s.
Spine	33 (40.74)	25 (75.76)	8 (24.24)	n.s.	14 (42.42)	7 (21.21)	12 (36.36)	n.s.
Pelvis	24 (29.63)	17 (70.83)	7 (29.17)	n.s.	6 (25.00)	8 (33.33)	10 (27.03)	n.s.
Head	36 (44.44)	2 (69.44)	11 (30.56)	n.s.	13 (36.11)	10 (27.78)	13 (36.11)	n.s.
Lung	41 (50.62)	29 (70.73)	12 (29.27)	n.s.	15 (36.59)	12 (29.27)	14 (34.15)	n.s.
Upper extremities	19 (23.46)	17 (89.47)	2 (10.53)	n.s.	6 (31.58)	5 (26.32)	8 (42.11))	n.s.
Lower extremities	30 (37.04)	22 (73.33)	8 (26.67)	n.s.	11 (36.67)	8 (26.67)	11 (36.67)	n.s.
Thoracic aorta	5 (6.17)	4 (80.00)	1 (20.00)	n.s.	0 (0.00)	2 (40.00)	3 (60.00)	n.s.
Liver	18 (22.22)	13 (72.22)	5 (27.78)	n.s.	5 (27.78)	8 (44.44)	5 (27.78)	n.s.
Spleen	25 (30.86)	18 (72.00)	7 (28.00)	n.s.	8 (32.00)	6 (24.00)	11 (44.00)	n.s.
Mesentery	12 (14.81)	7 (58.33)	5 (41.67)	n.s.	3 (25.00)	4 (33.33)	5 (41.67)	n.s.
Ileum	13 (16.05)	8 (61.54)	5 (38.46)	n.s.	3 (28.03)	5 (38.46)	5 (38.46)	n.s.
Intestine	12 (14.81)	8 (66.67)	4 (33.33)	n.s.	3 (25.00)	4 (33.33)	5 (41.67)	n.s.
Adrenal	11 (13.58)	9 (81.82)	2 (18.18)	n.s.	0 (0.00)	0 (0.00)	11 (100.00)	0.0005 ^3^
Retroperitoneum hematoma	68 (83.95)	52 (76.47)	16 (23.53)	n.s.	12 (17.65)	21 (30.88)	35 (51.47)	0.00005
Urinary bladder	4 (4.94)	4 (100.00)	0 (0.00)	n.s.	0 (0.00)	2 (50.00)	2 (50.00)	n.s.
Pancreas	11 (13.8)	5 (45.45)	6 (54.55)	0.0089	3 (27.27)	5 (45.45)	3 (27.27)	n.s.
Stomach	7 (8.64)	4 (57.14)	3 (42.86)	n.s.	2 (28.57)	2 (28.57)	3 (42.86)	n.s.

**Table 2:** Initial laboratory outcomes and comorbidities by sex and renal injury grades. ^3^—Pearson’s chi-square test.

		Sex			AAST Injury Grades			
	Overall	Male	Female	P Level	Minor N = 23	Moderate N = 21	Severe N = 37	P Level
**Initial laboratory; n (%)**								
GCS ≤ 8	23 (28.40)	15 (66.22)	8 (34.78)	n.s.	7 (30.43)	7 (30.43)	9 (39.13)	n.s.
PULSE > 100	26 (32.10)	19 (73.08)	7 (26.92)	n.s.	7 (26.92)	8 (30.77)	11 (42.31)	n.s.
HGB > 10 G/DL	61 (75.31)	53 (86.89)	8 (13.11)	0.0001 ^3^	19 (31.15)	18 (29.51)	24 (39.34)	n.s.
CREA > 110	36 (44.44)	27 (75.00)	9 (25.00)	n.s.	8 (22.22)	9 (25.00)	19 (52.78)	n.s.
K^+^ > 5.5	7 (8.64)	6 (85.71)	1 (14.29)	n.s.	0 (0.00)	2 (28.57)	5 (71.43)	n.s.
WBC >11.0 K/L	66 (81.48)	51 (77.27)	15 (22.73)	n.s.	17 (25.76)	17 (25.76)	32 (48.48)	n.s.
CRP > 100	19 (23.46)	12 (63.16)	7 (36.84)	n.s	1 (5.26)	3 (15.79)	15 (78.95)	0.0029 ^3^
GFR < 60	22 (27.16)	14 (63.64)	8 (36.36)	n.s.	7 (31.82)	5 (22.73)	10 (45.45)	n.s.
SBP < 90	11 (13.58)	10 (90.91)	1 (9.09)	n.s.	2 (18.18)	4 (36.36)	5 (45.45)	n.s.
**Initial laboratory; mean (SD)**								
GCS ≤ 8	5.22 (1.76)	5.38 (1.69)	5.13 (1.85)	n.s.	4.57 (1.99)	5.43 (1.81)	5.56 (1.59)	n.s.
PULSE > 100	119.5 (11.42)	117.7 (10.7)	120.2 (11.88)	n.s.	122.7 (9.4)	120.9 (16.1)	116.5 (8.5)	n.s.
HGB > 10 G/DL	12.79 (1.87)	11.81 (1.90)	12.93 (1.84)	n.s.	13.36 (2.06)	12.85 (1.86)	12.28 (1.65)	n.s.
CREA > 110	161.8 (64.4)	164.7 (74.3)	160.9 (62.3)	n.s.	156.8 (74.1)	164.7 (68.8)	162.6 (61.7)	n.s.
POTAS > 5.5	6.47 (0.71)	6.20 (0.0)	6.51 (0.76)	n.s.	-	6.50 (1.3)	6.46 (0.53)	n.s.
WBC >11.0 K/L	18.09 (6.08)	17.11 (5.55)	18.38 (6.25)	n.s.	18.78 (8.06)	18.02 (5.76)	17.76 (5.15)	n.s.
CRP > 100	190.6 (65.3)	171.9 (61.9)	201.6 (67.4)	n.s.	182.5 (-)	256.5 (96.7)	178.0 (55.2)	n.s.
GFR < 60	45.50 (5.84)	43.50 (15.57)	46.64 (14.60)	n.s.	46.0 (14.8)	40.0 (16.7)	47.9 (14.4)	n.s.
SBP < 90	82.37 (5.84)	82.00 (0.0)	82.41 (6.16)	n.s.	85.00 (1.41)	82.75 (5.62)	81.02 (7.43)	n.s.
**Comorbidities; n (%)**								
Hypertension	17 (20.99)	12 (70.59)	5 (29.41)	n.s.	1 (4.35)	5 (23.81)	11 (29.73)	n.s.
Chronic renal failure	10 (12.35)	6 (60.0)	4 (40.0)	n.s.	2 (8.70)	2 (9.52)	6 (16.22)	n.s.
Diabetes mellitus	14 (17.28)	9 (64.29)	5 (35.71)	n.s.	2 (8.70)	3 (14.29)	9 (24.32)	n.s.

**Table 3:** Type of management, event in hospital by sex, and renal injury grade.

		Sex			AAST Injury Grades			
	Overall	Male	Female	P Level	Minor N = 23	Moderate N = 21	Severe N = 37	P Level
**Management; n (%)**								
Surgical								
Up to 8 h after admission	22 (27.16)	18 (81.82)	4 (18.18)	n.s.	0 (0.00)	1 (4.76)	21 (55,76)	<0.001
Postponed	9 (11.11)	7 (77.78)	2 (22.22)	n.s.	0 (0.00)	1 (5.34)	8 (18.21)	<0.001
Sum	31 (38.27)	25 (80.65)	6 (19.35)	n.s	0 (0.00)	2 (10.18)	29 (98.34)	<0.001
Conservative								
Up to 8 h after admission	59 (72.84)	44 (74.58)	15 (25.42)	n.s.	23 (95.65)	20 (90.48)	16 (37.84)	<0.001
Sum	50 (61.73)	37 (74.00)	13 (26.00)	n.s.	23 (95.65)	19 (89.74)	8 (18.21)	<0.001
**Complications; n (%)**								
Dialyses	10 (12.35)	6 (9.68)	4 (21.05)	n.s.	3 (13.04)	3 (14.29)	4 (10.81)	n.s.
Acute kidney damage	10 (12.35)	6 (9.68)	6 (9.68)	n.s.	3 (13.04)	3 (14.29)	4 (10.81)	n.s.
Urinary tract infection	7 (8.64)	5 (8.06)	2 (10.53)	n.s.	3 (13.04)	2 (9.52)	2 (5.41)	n.s.
Acute respiratory distress syndrome	39 (48.15)	28 (45.16)	11 (57.89)	n.s.	13 (56.52)	11 (52.38)	15 (40.54)	n.s.
Deep vein thrombosis	12 (14.81)	9 (14.52)	3 (15.79)	n.s.	5 (21.74)	3 (14.29)	4 (10.81)	n.s.
Bedsores	10 (12.35)	6 (9.68)	4 (21.05)	n.s.	4 (17.39)	2 (9.52)	4 (10.81)	n.s.
Pneumonia	21 (25.93)	17 (27.42)	4 (21.05)	n.s.	9 (39.13)	7 (33.33)	5 (13.51)	n.s.
Sepsis	16(19.75)	12 (19.35)	4 (21.05)	n.s.	7 (30.43)	4 (19.05)	5 (13.51)	n.s.

**Table 4:** Logistic regression results (estimates and *p*. values) analyzing the individual influence of severity grades, associated injuries, co-morbidities, and demographic parameters on laboratory results.

		Laboratory Results							
		HGB > 10 G/DL		CREA > 110		GFR < 60		SBP < 90	
		Estimate	P Level	Estimate	P Level	Estimate	P Level	Estimate	P Level
**Associated injury**									
Pelvis		n.s.		1.92332	0.0310	n.s.		n.s.	
Thoracic aorta		n.s.		n.s.		n.s.		3.2246	0.0224
Spleen		n.s.		1.93466	0.0387	n.s.		n.s.	
Ileum		n.s.		n.s.		2.73962	0.0242	n.s.	
**Severity (vs. minor)**									
Major		−3.09978	0.04357	n.s.		n.s.		n.s.	
**Co-morbidities**									
Chronic kidney disease		n.s.		2.79799	0.0190	2.02230	0.0270	n.s.	
**Demographic parameters**									
Male gender		5.15392	0.00506	n.s.		n.s.		n.s.	
Age		−0.11174	0.03739	n.s.		n.s.		n.s.	

**Table 5:** Logistic regression results (estimates and *p*. values) analyzing the individual influence of severity grades, associated injuries, co-morbidities, and demographic parameters on laboratory results.

	Patient Complications							
	Urinary Tract Infection		Pneumonia		Sepsis		Death	
	Estimate	P Level	Estimate	P Level	Estimate	P Level	Estimate	P Level
**Associated injuries**								
Lower extremities	n.s.		1.9002	0.0114	n.s.		n.s.	
Pancreas	n.s.		n.s.		n.s.		2.93721	0.0469
**Co-morbidities**								
Chronic kidney disease	2.1313	0.0297	n.s.		2.4445	0.00619	n.s.	

## Data Availability

The data presented in this study are available on request from the Michał Kasperczak (project manager). The data are available due to the Polish law and the regulations of the Medical University of Poznań.
